# Clinical and Imaging Features of Non-Small Cell Lung Cancer with G12C KRAS Mutation

**DOI:** 10.3390/cancers13143572

**Published:** 2021-07-16

**Authors:** Markus Y. Wu, Eric W. Zhang, Matthew R. Strickland, Dexter P. Mendoza, Lev Lipkin, Jochen K. Lennerz, Justin F. Gainor, Rebecca S. Heist, Subba R. Digumarthy

**Affiliations:** 1 Department of Radiology, Division of Thoracic Imaging and Intervention, Massachusetts General Hospital, Boston, MA 02114, USA; mywu@mgh.harvard.edu (M.Y.W.); ewzhang@mgh.harvard.edu (E.W.Z.); dpmendoza@mgh.harvard.edu (D.P.M.); 2 Cancer Center, Department of Medicine, Massachusetts General Hospital, Boston, MA 02114, USA; matthewr_strickland@dfci.harvard.edu (M.R.S.); jgainor@mgh.harvard.edu (J.F.G.); rheist@partners.org (R.S.H.); 3 Center for Integrated Diagnostics, Department of Pathology, Massachusetts General Hospital, Boston, MA 02114, USA; llipkin@mgh.harvard.edu (L.L.); jlennerz@partners.org (J.K.L.)

**Keywords:** KRAS mutation, lung cancer, radiology, targeted therapy

## Abstract

**Simple Summary:**

KRAS G12C mutations are important oncogenic mutations in lung cancer that can now be targeted by allosteric small molecule inhibitors. We assessed the imaging features and patterns of metastases in these lung cancers compared to other mutated lung cancers. We found that KRAS G12C NSCLC has distinct primary tumor imaging features and patterns of metastasis when compared to those of NSCLC driven by other genetic alterations. These distinct imaging features may offer clues to its presence and potentially guide management in the future.

**Abstract:**

KRAS G12C mutations are important oncogenic mutations that confer sensitivity to direct G12C inhibitors. We retrospectively identified patients with KRAS+ NSCLC from 2015 to 2019 and assessed the imaging features of the primary tumor and the distribution of metastases of G12C NSCLC compared to those of non-G12C KRAS NSCLC and NSCLC driven by oncogenic fusion events (RET, ALK, ROS1) and EGFR mutations at the time of initial diagnosis. Two hundred fifteen patients with KRAS+ NSCLC (G12C: 83; non-G12C: 132) were included. On single variate analysis, the G12C group was more likely than the non-G12C KRAS group to have cavitation (13% vs. 5%, *p* = 0.04) and lung metastasis (38% vs. 21%; *p* = 0.043). Compared to the fusion rearrangement group, the G12C group had a lower frequency of pleural metastasis (21% vs. 41%, *p* = 0.01) and lymphangitic carcinomatosis (4% vs. 39%, *p* = 0.0001) and a higher frequency of brain metastasis (42% vs. 22%, *p* = 0.005). Compared to the EGFR+ group, the G12C group had a lower frequency of lung metastasis (38% vs. 67%, *p* = 0.0008) and a higher frequency of distant nodal metastasis (10% vs. 2%, *p* = 0.02). KRAS G12C NSCLC may have distinct primary tumor imaging features and patterns of metastasis when compared to those of NSCLC driven by other genetic alterations.

## 1. Introduction

The discovery of genetic drivers of lung cancer has led to a dramatic paradigm shift in therapeutic strategies for patients with advanced non-small cell lung cancer (NSCLC). Historically, cytotoxic chemotherapy was the mainstay of treatment for advanced-stage disease; however, small molecules targeting genetic alterations in oncogenic drivers, such as epidermal growth factor receptor (EGFR) and anaplastic lymphoma kinase (ALK) [1,2], have produced improved clinical outcomes. Currently, eight distinct oncogenic drivers have corresponding FDA-approved targeted therapies available for use in NSCLC, and efforts are ongoing to identify and drug new targets.

Kristen rat sarcoma viral oncogene (KRAS) is detected in approximately 25% of non-squamous NSCLC, and is associated with white race and a positive smoking history [3,4]. KRAS cycles between RAS-guanosine triphosphate (GTP)-bound active and RAS-guanosine diphosphate (GDP)-bound inactive states. Until recently, almost no drugs were able to directly target KRAS due to its high affinity for GTP/GDP and the lack of a clear binding pocket [5]. Recent advances in the development of covalent inhibitors of G12C, which take advantage of the cysteine 12 residue to lock the protein in its inactive GDP bound conformation, have demonstrated promising signals of activity in clinical trials and are poised to dramatically change the treatment landscape for patients with KRAS G12C mutations owing to the relative high frequency of this alteration (approximately 13% of lung cancer) [6,7,8]. Indeed, one direct G12C inhibitor, sotorasib, recently gained approval by the United States Food and Drug Administration for treatment of patients with KRAS G12C-mutated NSCLC who have received at least one prior line of therapy [9].

There are ongoing efforts to define distinct biological differences across oncogenic drivers, which may result in differences in metastatic potential or prognosis. Prior studies have reported distinct imaging findings that may be seen in NSCLC harboring specific genetic alterations, including *ALK*, *ROS1*, *RET*, and *EGFR* [10,11,12,13,14,15,16,17]. It remains unknown whether there are distinct radiologic features associated with KRAS G12C NSCLC that may provide insights into biology and metastatic potential of this driver mutation, which may in turn inform surveillance and imaging strategies. In this study, we performed a retrospective analysis to determine the imaging and clinical features of G12C KRAS NSCLC compared to those of non-G12C KRAS mutations and to other genetic alterations with established targeted therapies.

## 2. Materials and Methods

### 2.1. Patient Selection and Data Extraction

**Figure 1:**
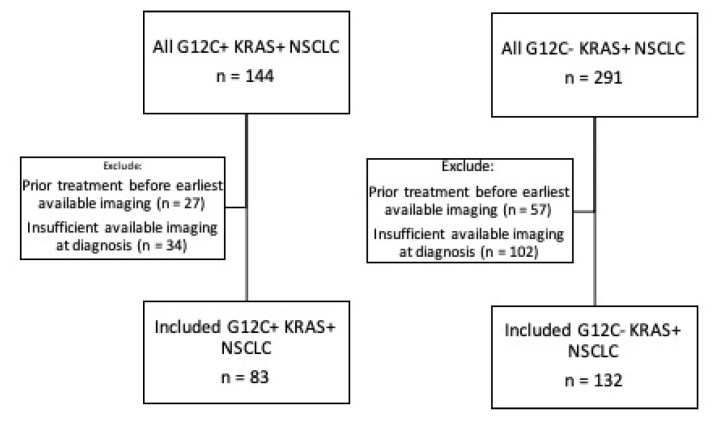
Inclusion and exclusion of patient cohorts.

This study was performed under an institutional review board–approved protocol (Partners Human Research Protocol #2019P000198). It is standard clinical practice at our institution to perform comprehensive tumor molecular profiling for all newly diagnosed patients with advanced non-small cell lung cancer. A retrospective database query of our laboratory information management system identified all NSCLC patients (May 2015–October 2019) with KRAS mutations, including the G12C KRAS variant, identified at our institution. Inclusion in our study required the availability of full imaging scans for initial staging at diagnosis, prior to any treatment for lung cancer, performed either at our institution or at an outside hospital with images uploaded into our institutional PACS (Visage 7, Visage Imaging, San Diego, CA, USA). The patient inclusion and exclusion process are summarized in Figure 1.

Electronic medical records were retrospectively reviewed to extract clinicopathologic data, including age, sex, race/ethnicity (self-identified), smoking history, tumor histologic findings, and disease stage identified at initial presentation, in accordance with the seventh edition of the American Joint Committee on Cancer’s Cancer Staging Manual. For the comparison groups of NSCLC patients driven by other genetic alterations, we used data from our institutional database from earlier publications on NSCLC driven by RET (rearranged during transfection), ALK, ROS1 (ROS proto-oncogene 1), and EGFR genetic alterations (variables were only included if data were available across every mutation group) [10,11,14,15,16,17].

### 2.2. Pathologic and Genetic Analysis

The histopathology and genetic analysis were performed on formalin-fixed paraffin-embedded tissue samples from either the primary lung tumor or metastases obtained by surgical resection, image-guided biopsy, or bronchoscopy. KRAS mutation status was determined using an institutional, targeted, next-generation sequencing panel (SNaPshot^®^ platform, Applied Biosystems, Foster City, CA, USA). This system detects single nucleotide polymorphisms and assesses single nucleotide variants and insertions/deletions in 104 known cancer genes [18,19]. The assay has been implemented clinically and all testing was performed in a clinical laboratory improvement amendments (CLIA) certified laboratory.

### 2.3. Imaging Review and Analysis

For each patient, baseline, pre-treatment imaging studies were analyzed. Required imaging studies included, at minimum, computed tomography (CT) scans of the chest, abdomen, and pelvis with or without concurrent fluorodeoxyglucose (FDG)-positron emission tomography (PET), and CT and/or magnetic resonance imaging (MRI) of the brain. The CT examinations of the body (chest, abdomen, and pelvis) were performed on multidetector-row CT scanners of multiple vendors with helical acquisition mode, automatic exposure control, tube potential 100–120 kV, slice thickness of 1–2.5 mm for chest and 5 mm for abdomen, with sagittal and coronal reformatted images. The brain MR and/or CT images were reviewed for the presence of intracranial metastases. The 18-FDG PET images, when available, were reviewed to assess for metabolic activity and were correlated with CT images. All imaging was performed at our institution or at another facility with the images subsequently uploaded to our PACS (Visage 7, Visage Imaging, San Diego, CA, USA). A board-certified radiologist specializing in lung cancer imaging and a thoracic imaging fellow retrospectively reviewed all imaging concurrently. Findings were determined and recorded by consensus.

The primary tumor, when identifiable, was evaluated for the following features: size, lobe, axial location (i.e., central versus peripheral), density (i.e., solid versus subsolid), and the presence of other features, including air bronchograms, cavities, calcifications, and lymphangitic carcinomatosis. When multifocal lung cancer was present, the size and location of the dominant tumor were recorded. Lymph nodes were determined to be malignant in the setting of a positive histologic finding, increased uptake on FDG PET, interval growth on follow-up imaging, or a combination of these findings, and they were recorded as ipsilateral or contralateral and as hilar, mediastinal, supraclavicular, or distant (e.g., cervical, axillary, or intra-abdominal). All indeterminate lymph nodes were presumed to be benign. The sites examined for metastases included the lungs, pleura, pericardium, liver, adrenal glands, other visceral organs (e.g., spleen, kidney, and other such organs), bones, subcutaneous soft tissues, and brain. Brain metastases were identified using CT or MRI of the brain and were classified as solitary or multiple, and infra- or supratentorial. Bone metastases were classified as lytic or sclerotic types. Assessment of bone metastasis was done at sites without evidence of fracture.

### 2.4. Statistical Analysis

Direct comparisons were performed between patients with KRAS G12C and non-G12C KRAS tumors. Patients with G12C tumors were also compared with patients with fusion rearrangements (RET+, ALK+, ROS1+), or EGFR+ using pairwise comparisons in order to preserve power given the modest sample sizes. Categorical variables were compared using either chi-squared or Fisher’s exact tests, while continuous variables were compared using Wilcoxon rank-sum test. *p*-values were reported for two-sided tests. A *p*-value of <0.05 was deemed significant. A multivariable analysis was performed to determine the variables that can distinguish between G12C and non-G12C KRAS NSCLC with candidate predictors chosen according to a *p* < 0.20 on a univariate analysis. Statistical analysis was performed using R Statistical Software (Version 4.03; R Foundation for Statistical Computing, Vienna, Austria).

## 3. Results

### 3.1. G12C KRAS vs. Non-G12C KRAS

**Figure 2:**
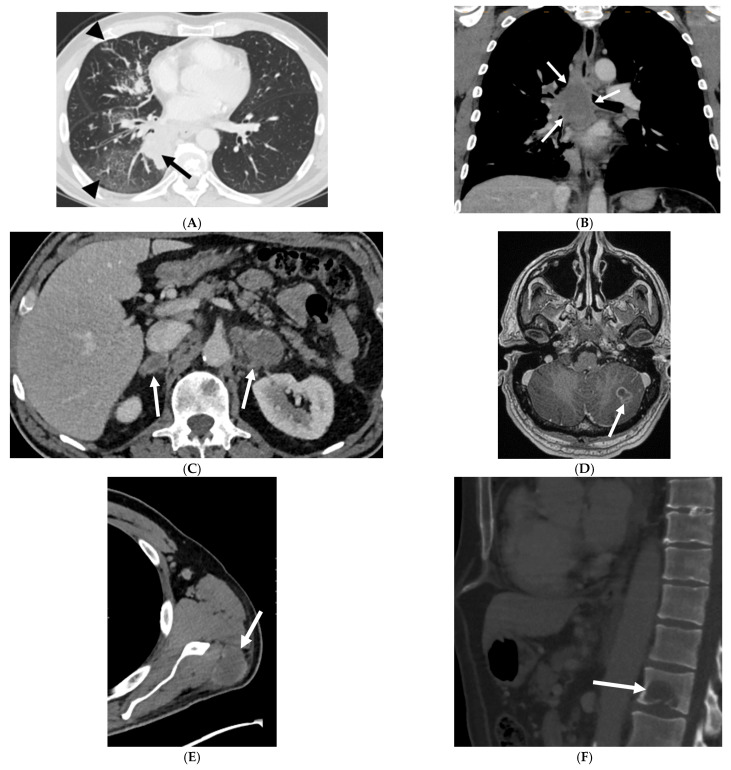
Representative imaging features in a 64-year-old male prior smoker with G12C KRAS NSCLC. Pretreatment CT images show a solid mass in the right lower lobe ((**A**), black arrow) and associated septal and peribronchial thickening consistent with lymphangitic carcinomatosis ((**A**), black arrowheads). There was extensive mediastinal and hilar lymphadenopathy ((**B**), white arrows), bilateral adrenal metastases ((**C**), white arrows), brain metastasis ((**D**), white arrow), soft tissue metastasis ((**E**), white arrow), and a lytic osseous metastasis of the first lumbar vertebral body ((**F**), white arrow).

This study included 215 patients with NSCLC harboring KRAS gene mutations (G12C: 83; non-G12C: 132). The baseline characteristics of these patients are summarized in Table 1. All tumors included in the study are adenocarcinomas. Patients with G12C KRAS NSCLC were more likely to be prior or current smokers (*N* = 83 (100%) vs. *N* = 125 (95%); *p* = 0.033) compared with patients with non-G12C KRAS NSCLC. Stratification by age, sex, or ethnicity at initial diagnosis did not reveal significant differences across KRAS genotypes. Table 2 summarizes the comparison of the two groups with respect to the CT features of the primary tumor. G12C KRAS tumors were more likely to have cavitation than were non-G12C KRAS tumors (13% vs. 5%, *p* = 0.04). Otherwise, no significant differences were observed between the two groups with respect to primary tumor size, tumor lobar and axial location, multifocality, or density. The majority of the primary tumors in both groups were solid in density (Figure 2A; representative images from a single patient), with infrequent incidence of air bronchogram, cavitation, and calcifications. The comparison of metastatic patterns among the two tumor genotypes is summarized in Table 3. A higher frequency of lung metastasis was noted at initial diagnosis in G12C KRAS NSCLC compared to that of non-G12C KRAS NSCLC (38% vs. 21%; *p* = 0.043). The incidence of pleural, lymphangitic, or pericardial spread were comparable across the two groups. Both cohorts had a high incidence of extrathoracic metastases (detected in 82–83% of patients), involving distant lymph nodes, liver, adrenal glands (Figure 2C), brain (Figure 2D), soft tissues (Figure 2E), and bone (Figure 2F). On multivariate analysis, there was no statistical significance for any variables to differentiate G12C from non-G12C KRAS NSCLC (*p* = 0.137).

### 3.2. G12C KRAS to Other Non-KRAS Oncogenic Genetic Alterations

#### 3.2.1. Patient Characteristics

The clinical features of the G12C KRAS subgroup were compared to those of patients with tumors harboring fusion rearrangements (*N* = 215, including RET, ALK, and ROS1 rearrangements), and EGFR mutation (*N* = 117). The clinical characteristics of these patients are summarized in Table 4. Patients in the G12C KRAS group were more likely to be Caucasian (99% vs. 77–80%; *p* = 0.001) and more likely to be a prior or current smoker (100% vs. 28–38%; *p* = 0.0001) compared to those in all the other mutation groups. 

#### 3.2.2. Imaging Features of the Primary Tumor

Table 5 summarizes the comparison of the G12C KRAS group to those of all the other mutations with respect to the CT features of the primary tumor. With regards to tumor density, the G12C KRAS group was less likely to be solid than was the fusion rearrangement group (89% vs. 97%; *p* = 0.004). The G12C KRAS group was less likely to demonstrate an air bronchogram than was the EGFR+ group (5% vs. 28%; *p* = 0.0001). The G12C KRAS group was more likely to have cavitation (13% vs. 3%; *p* = 0.005) and tumoral calcification (4 vs. 0%; *p* = 0.005) than was the fusion rearrangement group.

#### 3.2.3. Patterns of Metastases

**Figure 3:**
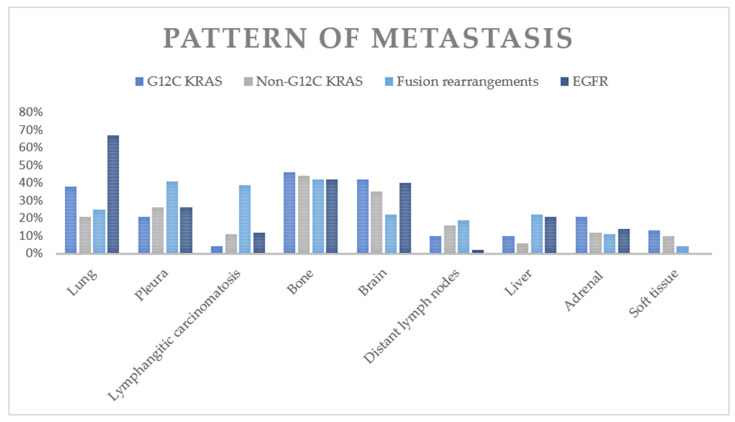
Frequencies of various metastatic sites in G12C KRAS+ NSCLC and in other genetic alterations.

The comparison of metastatic patterns between patients with G12C KRAS tumors and patients with tumors containing other non-KRAS mutations is summarized in Table 6 and Figure 3. The G12C KRAS group had a lower frequency of intrathoracic metastasis than did the fusion rearrangement group (52% vs. 75%, *p* = 0.002) and EGFR+ group (52% vs. 82%, *p* = 0.0001). The G12C group had a lower frequency of lung metastasis than did the EGFR mutation group (38% vs. 67%, *p* = 0.0008). The G12C group had a lower frequency of pleural metastasis than did the fusion rearrangement group (21% vs. 41%, *p* = 0.01). The G12C group had a lower frequency of lymphangitic carcinomatosis than did the fusion rearrangement group (4% vs. 39%, *p* = 0.0001).

With respect to extrathoracic metastasis, the G12C group had a higher frequency of brain metastasis than did the fusion rearrangement group (42% vs. 22%, *p* = 0.005). The G12C group had a higher frequency of distant lymph node metastasis (10% vs. 2%, *p* = 0.02) than did the EGFR+ group. The G12C group also had a higher frequency of soft tissue metastasis than did the fusion rearrangement group (13% vs. 4%, *p* = 0.044) and the EGFR+ group (13% vs. 0%, *p* = 0.0004).

## 4. Discussion

Recently, the KRAS G12C mutation has been identified as a targetable oncogenic mutation in NSCLC with promising investigational agents currently in clinical trials [5,6,20,21], and one agent, sotorasib, recently receiving accelerated approval by the US FDA [9]. To the best of our knowledge, this is the first systematic assessment of the imaging features of the primary tumor and patterns of metastasis in NSCLC with the G12C KRAS mutation. We found that G12C and non-G12C KRAS NSCLCs share many similar clinical and radiologic features. Some differences found on single variate comparison but not on multivariate comparison include that the G12C KRAS group had more current or prior smokers, was more likely to have a cavitary primary tumor, and had a higher frequency of lung metastasis. Compared to NSCLC with fusion rearrangement mutations, the G12C KRAS group demonstrated a lower frequency of pleural metastasis and lymphangitic carcinomatosis and a higher frequency of brain and soft tissue metastasis. Compared to the EGFR+ group, the G12C KRAS group had a lower frequency of lung metastasis and a higher frequency of distant lymph node and soft tissue metastasis.

With therapies targeting G12C KRAS mutations now entering standard clinical practice, differentiating G12C KRAS NSCLC from non-G12C KRAS NSCLC has important prognostic and therapeutic implications. Sensitive and specific predictions of oncogene-driven lung cancer based on clinical and imaging characteristics can be beneficial in terms of accelerated detection of a rational target, more streamlined triaging of patients for genomic testing, and prioritizing certain genomic testing in cases with limited tissue. However, our findings suggest a considerable overlap in terms of both clinical and imaging features between G12C KRAS and non-G12C KRAS NSCLC. Clinically, we found that patients with G12C KRAS NSCLC were more often white and more commonly found to be current or prior smokers, consistent with prior literature [22,23].

One potential differentiating imaging feature between G12C and non-G12C KRAS NSCLC was the increased frequency of cavitation within the primary tumors of G12C KRAS NSCLC, although the absolute frequency is still rare in both groups and no significant difference was found on multivariate analysis. The presence of cavitation in primary lung cancers has previously been associated with worse prognosis and has long been postulated to be related to rapid tumor growth outstripping the local vascular supply [24,25,26]. Nonetheless, future molecular studies with larger sample sizes are needed to elucidate the biological and pathophysiologic differences behind G12C and non-G12C KRAS mutations and how these differences may manifest as perceptible radiologic findings.

Notably, more imaging differences were found comparing G12C KRAS NSCLC with other genetic alterations seen in NSCLC. These differences may provide value for clinicians in diagnosing, treating, and following lung cancer patients. Compared to EGFR+ NSCLC, we found that G12C NSCLC were less likely to have lung metastases and air bronchograms. The increased prevalence of diffuse or miliary metastases in EGFR+ mutations has been reported in several studies [14,27], and their presence can potentially aid clinicians in considering EGFR+ over KRAS+ mutations.

With respect to patterns of metastasis, the most common sites of metastases in advanced G12C KRAS NSCLC in our cohort were the bones (46%), brain (42%), and lungs (38%). In contrast, the fusion rearrangement group was heterogeneous with an overall lower rate of brain metastases (22%). Yang et al. demonstrated that KRAS mutations were risk factors for brain metastasis in male patients with lung adenocarcinomas [28]. The higher incidence of brain metastases in the G12C KRAS subgroup is clinically relevant and suggests these patients may benefit from closer monitoring for neurological signs and/or symptoms as well as highlights the need for therapeutic agents that can reliably penetrate and remain active beyond the blood–brain barrier.

While bone metastases were found in similar frequency across NSCLC genotypes, differences were found in terms of the characteristics of the bone metastasis. All osseous metastases in the G12C KRAS group were lytic in contrast to the higher frequency of sclerotic metastasis in the RET+, ROS1+, and ALK+ NSCLC groups [10,17,27]. Osteolytic lesions are caused by factors that stimulate osteoclast activity and bone resorption, including parathyroid hormone-related peptide and interleukins, as well as factors that inhibit osteoblastic activity such as protein Dickkopf-1 [29]. Bone metastases are a significant cause of morbidity from pain and fractures reducing quality of life. The presence of bone metastases in KRAS+ advanced lung adenocarcinoma has been shown to be associated with worse outcomes [30].

Patients with G12C NSCLC also exhibit higher frequency of soft tissue metastasis (13%) compared to those with fusion rearrangements (4%) and EGFR mutations (0%), consistent with prior reports [31]. To our knowledge, this is the first study to report a relatively higher incidence of soft tissue metastasis in G12C KRAS NSCLC. This is potentially significant because soft tissue metastasis, though rare, is associated with poor prognosis and worse response to treatment in advanced lung cancer [32,33].

Our study has several limitations. Due to its retrospective, single-institution nature, the findings may not be generalizable to larger populations. While our patient cohort represents the largest group of G12C NSCLC patients to date, the sample size is still relatively small and thus limits the statistical power to detect significant differences across molecular subgroups. Furthermore, the modest sample size may also limit the statistical power of the multivariable analysis adjusting for confounding factors. In addition, co-mutations such as TP53, and STK11 are relatively frequent in KRAS mutants and that co-mutation status can determine prognosis and treatment response [34,35]. Our cohort does not have enough statistical power to differentiate between these co-mutations and future studies are warranted for further characterization. The imaging findings were determined by consensus, not by independent review, which was another inherent limitation. Finally, although our findings suggested distinct imaging features that might be helpful in distinguishing G12C NSCLC from non-G12C NSCLC, elucidation of the biologic mechanisms underlying these differences for metastatic tropism or primary tumor characteristics was beyond the scope of this study. Despite these limitations, our findings add to the growing understanding of the clinical and imaging features of G12C NSCLC, and it is the only study comparing G12C NSCLC to NSCLC with targetable driver oncogenes.

## 5. Conclusions

To our knowledge, this is the first study to date to assess the imaging features and metastatic patterns of G12C KRAS NSCLC. Our findings suggest that G12C KRAS tumors have certain imaging features and patterns of metastasis that are distinct compared with those of other oncogenic mutations in NSCLC. Although these radiologic features cannot substitute for appropriate molecular testing to detect oncogenic driver alterations, they may nevertheless assist in selecting those patients who are most likely to benefit from expedited genotyping or repeat testing after receiving an initial nondiagnostic result. Additionally, our data have value for training deep learning models as we move towards a future with increasing integration of artificial intelligence in diagnostic and therapeutic clinical practice. Ultimately, more work is needed to determine the role of imaging biomarkers in predicting the presence of certain oncogenic drivers.

## Figures and Tables

**Table 1:** Clinical features of G12C KRAS and non-G12C KRAS NSCLC.

Clinical Features	G12C KRAS	Non G12C KRAS	
	*N* = 83	*N* = 132	*p*-Value
Age			
Median	67	69	0.806
Range	(44–91)	(42–92)	
Sex			
Female	48	70	0.491
	58%	53%	
Male	35	62	
	42%	47%	
Race			
Caucasian	82	128	0.57
	99%	97%	
Asian	1	1	
	1%	1%	
Other	0	2	
	0%	2%	
Smoking history			
Never	0	7	0.03318
	0%	5%	
Prior/current	83	125	
	100%	95%	
Stage			
I	12	20	0.6141
	14%	15%	
II	6	12	
	7%	9%	
III	17	18	
	21%	14%	
IV	48	82	
	58%	62%	

**Table 2:** Imaging features of primary tumors in G12C and non-G12C KRAS positive NSCLC.

Imaging Feature	G12C KRAS	Non-G12C KRAS	
	*N* = 83	*N* = 132	*p*-Value
Tumor size (mm)			
Median	41	38	0.5876
Range	(7–96)	(9–121)	
Tumor location			
Right upper lobe	27 (33%)	38 (29%)	0.71
Right middle lobe	4 (5%)	11 (8%)	
Right lower lobe	22 (26%)	27 (20%)	
Left upper lobe	14 (17%)	29 (22%)	
Lingula	1 (1%)	3 (2%)	
Left lower lobe	15 (18%)	24 (19%)	
Multifocal tumor	17 (21%)	22 (17%)	0.48
Axial location			
Inner	33 (40%)	47 (36%)	0.3305
Middle	4 (5%)	2 (2%)	
Outer	41 (49%)	69 (52%)	
All	5 (6%)	14 (10%)	
Tumor density			
Solid	74 (89%)	118 (89%)	0.997
Ground glass	2 (2%)	3 (2%)	
Mixed	7 (9%)	11 (9%)	
Tumor margin			
Smooth	47 (57%)	93 (70%)	0.052
Lobulated	19 (23%)	15 (11%)	
Spiculated	17 (20%)	24 (19%)	
Chest wall invasion	1 (1%)	1 (1%)	0.739
Air bronchogram	4 (5%)	7 (5%)	0.875
Cavitation	11 (13%)	7 (5%)	0.04
Tumoral calcification	3 (4%)	1 (1%)	0.131

**Table 3:** Pattern of metastases in G12C and non-G12C KRAS positive NSCLC.

Metastatic Site	G12C KRAS	Non-G12C KRAS	
	*N* = 48	*N* = 82	*p*-Value
Intrathoracic	25	36	0.656
	52%	44%	
Lung	18	17	0.043
	38%	21%	
Pleural	10	21	0.433
	21%	26%	
Lymphangitic carcinomatosis	2	9	0.153
	4%	11%	
Extrathoracic	40	67	0.714
	83%	82%	
Bone	22	36	0.902
	46%	44%	
Brain	20	29	0.717
	42%	35%	
supratentorial only	10 (12%)	11 (8%)	0.412
infratentorial only	2 (2%)	8 (6%)	
both supratentorial and infratentorial	9 (11%)	10 (8%)	
Distant lymph nodes	5	13	0.324
	10%	16%	
Liver	5	5	0.448
	10%	6%	
Adrenal	10	10	0.272
	21%	12%	
Soft tissue	6	8	0.735
	13%	10%	

**Table 4:** Clinical features of G12C KRAS and non-KRAS NSCLC.

Clinical Features	G12C	Fusion Rearrangements	EGFR	G12C vs. Fusion Rearrangements	G12C vs. EGFR
	*N* = 83	*N* = 215	*N* = 117	*p* Value	*p* Value
Sex					
Female	48	129	81	0.7279	0.1019
	58%	60%	69%		
Male	35	86	36		
	42%	40%	31%		
Race					
Caucasian	82	165	94	0.0001	0.0001
	99%	77%	80%		
Asian	1	32	15		
	1%	15%	13%		
Other	0	18	8		
	0%	8%	7%		
Smoking history					
Never	0	155	72	0.0001	0.0001
	0%	72%	62%		
Prior/current	83	60	45		
	100%	28%	38%		

**Table 5:** Imaging features of the primary tumor in G12C KRAS and other targetable genetic alteration-driven NSCLC.

Imaging Feature	G12C	Fusion Rearrangements	EGFR	G12C vs. Fusion Rearrangements	G12C vs. EGFR
	*N* = 83	*N* = 215	*N* = 117	*p* Value	*p* Value
Tumor location				0.0617	N/A
Right upper lobe	27 (33%)	46(21%)			
Right middle lobe	4 (5%)	26 (12%)			
Right lower lobe	22 (27%)	51 (24%)			
Left upper lobe	14 (17%)	38 (18%)			
Lingula	1 (1%)				
Left lower lobe	15 (18%)	54 (25%)			
Multifocal	17 (21%)				
Axial location				0.0192	0.0022
Inner	33 (40%)	115 (53%)	54 (46%)		
Middle	4 (5%)				
Outer	41 (49%)	100 (47%)	75 (35%)		
All	5 (6%)		29 (25%)		
Tumor density				0.0044	0.2348
Solid	74 (89%)	209 (97%)	104 (89%)		
Ground glass	2 (2%)				
Mixed	7 (8%)	6 (3%)	13 (11%)		
Tumor margin					
Smooth	47 (57%)				
Lobulated	19 (23%)				
Spiculated	17 (20%)				
Air bronchogram	4 (5%)	12 (6%)	28 (28%)	0.79486	0.0001
Cavitation	11 (13%)	2 (3%)	13 (13%)	0.0051	0.6643
Calcification	3 (4%)	0	1 (1%)	0.0051	0.3092

**Table 6:** Pattern of metastases in G12C KRAS and other targetable genetic alteration-driven NSCLC.

Metastatic Site	G12C	Fusion Rearrangements	EGFR	G12C vs. Fusion Rearrangements	G12C vs. EGFR
	*N* = 48	*N*=158	*N* = 117	*p* Value	*p* Value
Intrathoracic	25	119	96	0.00214	0.0001
	52%	75%	82%		
Lung	18	40	78	0.101	0.0008
	38%	25%	67%		
Pleural	10	65	31	0.01046	0.5529
	21%	41%	26%		
Lymphangitic carcinomatosis	2	62	14	0.0001	0.1551
	4%	39%	12%		
Pericardium	0	5	0	0.2113	>0.999
	0%	3%	0%		
Extrathoracic	40	111	84	0.07346	0.1645
	83%	70%	72%		
Bone	22	67	49	0.67488	0.7297
	46%	42%	42%		
Brain	20	34	47	0.00544	0.8633
	42%	22%	40%		
Distant lymph nodes	5	30	2	0.16758	0.0226
	10%	19%	2%		
Liver	5	34	24	0.8544	0.1758
	10%	22%	21%		
Adrenal	10	17	16	0.0703	0.2503
	21%	11%	14%		
Soft tissue	6	7	0	0.0444	0.0004
	13%	4%	0%		

## Data Availability

Data available on request due to privacy restrictions.
